# Gravimetry in sweating assessment in primary hyperhidrosis and healthy individuals

**DOI:** 10.1007/s10286-013-0201-2

**Published:** 2013-06-13

**Authors:** Tomasz J. Stefaniak, Monika Proczko

**Affiliations:** Department of General, Endocrine and Transplant Surgery, Medical University of Gdansk, 17 Smoluchowskiego St., 80-211 Gdańsk, Poland

**Keywords:** Hyperhidrosis, Palmar, Axillary, Sweating, Gravimetry

## Abstract

**Objective:**

Though hyperhidrosis is generally considered a subjectively perceived disease, it seems more and more doubtful that merely subjective evaluation is sufficient to qualify the patient to surgery. The aim of this study was to develop further gravimetry as a method of evaluation of sweating intensity and determination of the applicability of it in post-operative follow-up of primary hyperhidrosis (PHH) patients.

**Methods:**

Total of 1,485 gravimetry assays has been performed in 343 patients treated for hyperhidrosis and in 220 healthy volunteers. In all of the subjects the measurements were taken from four localizations (face, hands, armpits and trunk) and normalized by body surface of the participant. The measurements were taken twice for every participant to obtain test–retest correlations. Mean values and standard deviations (SD) have been evaluated and on that basis reference values were quantified. Thresholds for diagnosis of hyperhidrosis were quantified on the basis of normal distribution theory as healthy population mean +2 SD.

**Results:**

In healthy volunteers, mean value of gravimetrically evaluated intensity of sweating were: facial: 19.15 ± 14.97 mg/min/m^2^, palmar: 18.49 ± 14.06 mg/min/m^2^, axillary: 42.39 ± 47.08 mg/min/m^2^ and plantar: 15.77 ± 16.87 mg/min/m^2^. Thresholds for diagnosis of hyperhidrosis were quantified, respectively as: 49, 46, 136 and 50 mg/min/m^2^. The overall test–retest correlation was 0.71.

**Interpretation:**

Gravimetry is easy, reproducible and fast method of evaluation of sweating. The reference values are stable and can serve as a qualifying and follow-up tool for evaluation of the patients with PHH in any localization.

## Introduction

Though hyperhidrosis is generally considered a subjectively perceived disease, it seems more and more doubtful that merely subjective evaluation is sufficient to qualify the patient to surgery [1]. Vapometry provides valuable and reliable method of assessment, nevertheless, it is not possible to be performed it in multiple localizations (face, hands, armpits, trunk, feet) in more than one patient at the same time [2]. Gravimetry is a simple, cheap and fast method of objective evaluation of sweating, and seems to be very promising [3–5].

The aim of this study was further development of gravimetry as a method of evaluation of sweating intensity and determination of the applicability of it in post-operative follow-up of primary hyperhidrosis (PHH) patients.

## Participants and methods

1,045 gravimetry full assays (obtained from five different areas, together 5,225 single measurements) has been performed in 343 patients evaluated for suspicion of PHH, and during the follow-up visits of 229 patients that have been treated with videothoracoscopic R3-4 thoracoscopic sympathotomy by clipping in our institution as described before [6]. Apart from that 440 measurements (every measurement in four areas, together 1,760 single measurements) were obtained from 220 healthy volunteers (medical students). In all of the subjects the measurements were taken from four localizations (face, hands, armpits and trunk). The evaluation was taken twice in 3 months period of the post-sympathectomy follow-up (follow-up patients) and in 1 week (volunteers) to obtain test–retest measurements. The volunteers filled an anonymous questionnaires (marked by numbers to be combined with gravimetric results and retest scores) on their subjective perception of the intensity of their sweating.

Median age of the patients was 29 years (16–72), median age of the volunteers was 24 years (21–28). The male:female ratio in the group of patients was 0.48:1, in the group of students—0.37:1. Neither age distribution, nor gender ratio statistically differentiated the groups. The percentage of students claiming that they are characterized by an increased sweating was 3.1 %. None of them considered the sweating a significant problem or searched for surgical help.

Gravimetric assay have been performed similarly to the procedure described by Heckmann et al. [3] and Hund et al. [4]. In short, after 15 minutes’ rest in sitting position, the patients were invited into the air-conditioned measure room in standardized temperature (24–25 °C) and humidity 15–17 %. A standard small cotton gauze pad has been weighted on a precise (*d* = 0.5 mg) weight scale (Radwag, Poland—scale type WPS 110/C/S, Poland). Then the pad was given to the participant who was asked to wipe carefully the area under evaluation. The procedure in each localization lasted 1 min. Then the pad was weighted again and a difference was calculated. To avoid a bias associated with different body area of participants, the difference was standardized by division by body area calculated with the height of the participant (according to Mosteller, body area [m^2^] = 0.01667 × height [cm]^0.5^ × mass [kg]^0.5^) [7].

The stress stimulus was also standardized by the same description of surgical procedure and possible complications. For the control group, the stress stimulus was also standardized. It was a detailed description of very complex procedure of a final exam in surgery, that the participants were about to face.

The study protocol was approved by the local ethical committee of the Medical University of Gdansk, Poland.

## Results

Mean gravimetric intensity of sweating in PHH patients prior to surgery was 24.49 ± 45.64 mg/min/m^2^ for facial, 153.37 ± 160.39 mg/min/m^2^ for palmar, 66.23 ± 56.18 mg/min/m^2^ for axillary and 31.24 ± 72.97 mg/min/m^2^ for abdomino-lumbar localizations. After surgery it was 14.48 ± 11.64 mg/min/m^2^ for facial, 13.77 ± 17.65 mg/min/m^2^ for palmar, 23.63 ± 24.56 mg/min/m^2^ for axillary and 28.86 ± 56.05 mg/min/m^2^ for abdomino-lumbar localizations. In healthy volunteers, mean value of gravimetrically evaluated intensity of sweating was, respectively, 19.15 ± 14.97, 18.49 ± 14.06, 42.39 ± 47.08 and 15.77 ± 16.87 mg/min/m^2^.

The overall test–retest correlation was 0.71. Test–retest values measured in volunteers for different localizations were: facial 0.64, palmar 0.54, axillary 0.84 and abdomino-lumbar 0.70. In follow-up patients it was, respectively, 0.82, 0.81, 0.79 and 0.66 (only follow-up results were included in test–retest evaluation). Mean population values of gravimetry were calculated for four different areas. To obtain threshold for diagnosis of hyperhidrosis, double standard deviation was added to the mean.

In normal distribution, it is calculated that 95.5 % of observations is within mean ±2 SD. Considering that only the upper limit of normative value is important for diagnosing hyperhidrosis, it can be calculated that 2.25 % of cases will be within the range higher than mean + 2 SD. This percentage is in accordance with epidemiological data on predominance of PHH in population, which has been reported to reach 2.8 % [1, 8]. The analysis of the questionnaires of the students confirmed that those who reached higher scores in gravimetric assay were also subjectively considering their sweating as increased. Nevertheless, none of them requested surgery.

Therefore, the threshold for diagnosis of PHH was calculated. The thresholds for four different areas are presented in Table 1.

**Table 1:** Reference values of sweating in different localizations evaluated with gravimetry

Localization	Mean raw value in 1-min test (mg/min)	Mean value in 1-min test divided by body surface (mg/min/m^2^)	Upper limit of acceptance defined as mean +2 × standard deviation (mg/min/m^2^)
Facial	30	19	49
Palmar	29	18	46
Axillary	61	42	136
Abdomino-lumbar	27	16	50

## Discussion

In this study, for the first time in worldwide literature, we presented reference values for gravimetric evaluation of intensity of sweating in four localizations (face, hands, armpits and abdomino-lumbar area) in PHH patients and in healthy individuals.

Adequate and objective evaluation of intensity of sweating plays an essential role in proper qualification to surgery and further, for reliable assessment of the results of intervention. In some anecdotal cases, despite obvious anhidrosis, the patients may still demand further treatment [9], which may arise from psychiatric conditions, such as body dysmorphic syndrome. In those patients, invasive treatment may lead not only to dissatisfaction, but also to severe psychiatric disturbances or even suicide.

In most studies presented in the literature, the authors concentrate on relative impact of different forms of treatment on gravimetrically measured sweating [3, 4, 10–19]. Mostly, such measurements were performed in dermatological settings and concerned the results of botox treatment [3, 4, 13–17, 19] or iontophoresis [12]. In those methods of treatment, associated with low level of transient complications or side effects, even less stringent approach to qualification will not cause long-term harm to the patients. Thanks to regular introduction of the quantitative evaluation of sweating prior and after surgery, it is possible to provide the patients with information on their sweating compared to the reference values. Due to that it was possible to confirm that the preoperative values of abdomino-lumbar sweating in the PHH patients are very low. The increase in sweating in this area is very often subjectively experienced by the patients. Nevertheless, in our study the post-op abdomino-lumbar sweat rate remained low and not above the reference value found in our control subjects. Although this is encouraging and will help reduce the perception of some patients of compensatory hyperhidrosis, it would be necessary to test post-op patients for exercise and heat induced abdomino-lumbar sweating before concluding our post-op patients did not have any compensatory hyperhidrosis.

In context of sympathectomy and decision to qualify the patient to this radical form of treatment or to reoperation, it seems essential to obtain also reference values.

## Conclusion

Gravimetry is easy, reproducible and fast method of evaluation of sweating. The reference values are stable and can serve as a qualifying and follow-up tool for evaluation of the patients with PHH in any localization.
